# Accuracy of MRI-CT registration in brain stereotactic radiotherapy: Impact of MRI acquisition setup and registration method

**DOI:** 10.1016/j.zemedi.2022.04.004

**Published:** 2022-05-25

**Authors:** Siti Masitho, Florian Putz, Veit Mengling, Lisa Reißig, Raphaela Voigt, Tobias Bäuerle, Rolf Janka, Rainer Fietkau, Christoph Bert

**Affiliations:** a Department of Radiation Oncology, Universitätsklinikum Erlangen, Friedrich-Alexander-Universität Erlangen-Nürnberg (FAU), Erlangen, Germany; b Department of Radiology. Universitätsklinikum Erlangen, Friedrich-Alexander-Universität Erlangen-Nürnberg (FAU), Erlangen, Germany; c Comprehensive Cancer Center Erlangen-EMN (CCC ER-EMN), Erlangen, Germany

**Keywords:** Magnetic-resonance imaging, Registration, Brain radiotherapy, Intracranial stereotactic radiotherapy, Patient immobilization

## Abstract

**Background:**

In MR-based radiotherapy (RT), MRI images are co-registered to the planning CT to leverage MR image information for RT planning. Especially in brain stereotactic RT, where typical CTV-PTV margins are 1-2 mm, high registration accuracy is critical. Several factors influence the registration accuracy, including the acquisition setup during MR simulation and the registration methods.

**Purpose:**

In this work, the impact of the MRI acquisition setup and registration method was evaluated in the context of brain RT, both geometrically and dosimetrically.

**Methods and Materials:**

MRI of 20 brain radiotherapy patients was acquired in two MRI acquisition setups (RT and diagnostic). Three different automatic registration tools provided by three treatment planning systems were used to rigidly register both MRIs and CT in addition to the clinical registration. Segmentation-based evaluation using Hausdorff Distance (HD)/Dice Similarity Coefficient and landmark-based evaluation were used as evaluation metrics. Dose-volume-histograms were evaluated for target volumes and various organs at risks.

**Results:**

MRI acquisition in the RT setup provided a similar head extension as compared to the planning CT. The registration method had a more significant influence than the acquisition setup (Wilcoxon signed-rank test, p<0.05). When registering using a less optimal registration method, the RT setup improved the registration accuracy compared to the diagnostic setup (Difference: Δ*MHD* = 0.16 mm, Δ*HD_P95_* = 0.64 mm, mean Euclidean distance (Δ*mEuD)* = 2.65 mm). Different registration methods and acquisition setups lead to the variation of the clinical DVH. Acquiring MRI in the RT setup can improve PTV and GTV coverage compared to the diagnostic setup.

**Conclusions:**

Both MRI acquisition setup and registration method influence the MRI-CT registration accuracy in brain RT patients geometrically and dosimetrically. MR-simulation in the RT setup assures optimal registration accuracy if automatic registration is impaired, and therefore recommended for brain RT.

## 1 Introduction

Magnetic resonance imaging (MRI) is one of the most important imaging modalities in radiotherapy (RT) and - compared to computed tomography (CT) - delivers superior soft-tissue contrast which enables accurate delineation of target volumes and organs at risk (OAR).

For the use of MRI in RT, it is crucial to pay extra attention to specific RT requirements. High accuracy is necessary especially for brain stereotactic RT, where a high dose with a steep gradient is delivered. The typically used clinical target volume (CTV) to planning target volume (PTV) margins are in the order of 1–2 mm and may even be less than 1 mm for intracranial stereotactic radiosurgery (SRS) [1], [2], [3], [4]. Further, a high dose of up to 24 Gy may be delivered in a single fraction to small volumes. Hence, these minimal margins require a corresponding high accuracy across the whole treatment planning chain. There are other possible factors that should be considered for the CTV-PTV margins for the intracranial SRS, such as possible tumor growth and tissue deformations due to the time interval and positioning differences between MRI/CT acquisition and treatment delivery, as well as registration inaccuracies.

The MR-based RT is based on the CT for treatment planning as well as on the cone-beam CT for daily positioning. In the MR-based RT, MRI is frequently acquired in external departments. The MRI images are then co-registered to the planning CT images, and so the contoured target volumes are brought onto the CT frame-of-reference. Due to the different acquisition sites and the time interval between the MRI and CT acquisition (±5 days), multiple uncertainties are introduced due to tumor growth and displacement as well as the differences in positioning between MRI acquisition and treatment delivery [5], [6]. One important uncertainty derives from the MRI-CT registration inaccuracy. The accuracy of the MRI-CT registration has been investigated in various studies. In 2010 it was reported in a multi-institutional study that an average registration inaccuracy of up to 2 mm was present in intracranial SRS [7]. Even though the CTV-PTV margin in SRS was set to <1 mm, the registration inaccuracy was not considered in the determination of the CTV-PTV margin [1].

The AAPM TG 132 report for image registration has listed the different sources of error in the MRI-CT registration [8]. The difference between the acquisition setup during MRI and CT scans can lead to registration inaccuracy. Patient CT scans are commonly acquired in the same setup as during irradiation (RT setup). It has been reported that different patient positioning for MRI may lead to different extension angle at the occipito-atlanto-axial joint complex, which in turn may affect the registration accuracy due to rotational errors or even non-rigid deformation of infratentorial tissues [9], [10], [11], [12]. In the RT setup for MRI, the patient is positioned similarly to during irradiation [13], [14]. In a consensus on MRI simulation for external beam RT planning by Paulson et al. [15], acquisition in the RT setup was generally recommended. Several radiotherapy institutions have implemented MRI acquisition in the RT setup, i.e., for prostate and head and neck patients, and reported an improvement of the registration accuracy of up to 3 mm. It should be considered, however, that more deformations occur in the pelvis and head and neck compared to the brain, and consequentially less influence on the registration accuracy of the brain is expected.

In our institution, an RT setup for MRI acquisition for brain patients is implemented, where the patient is positioned on an RT flat table couch and immobilized using an RT mask. A dedicated mask holder and a receiving coil setup were developed. The novel RT head setup was reported to deliver a comparable signal-to-noise ratio (SNR) to the standard diagnostic setup [16]. With the same patient acquisition setup during both MRI and CT, it is hypothesized that registration is facilitated and thus accuracy is improved.

Another source of registration inaccuracy is the choice of the registration method. The current clinical standard for MRI-CT registration for the brain is rigid registration. As a part of our clinical standard, MRI-CT registration is done manually in the treatment planning system (TPS) of choice, which involves meticulous adjustment of both images, followed by visual inspection in an iterative manual process. This method can be time-consuming depending on the physician's expertise. Additionally, this method can be prone to interobserver variabilities. Most commercial treatment planning systems provide an automatic image registration tool, which accelerates the process. Commonly, automatic registration for multimodality registrations is based on mutual information, where mutual information is defined as a measure of dependence between two images [17].

The automatic registration accuracy depends strongly on the optimization in the registration algorithm, which varies widely across different platforms. Optionally, additional manual adjustments can be done, for example by defining a volume of interest (VOI) prior to registration. The accuracy of automatic registration for the brain has been investigated in several studies [18], [19]. These studies are however limited to the evaluation of MRI acquired only in the diagnostic setup (without patient immobilization) and did not evaluate the MRI acquired in the RT setup.

Thus, this work presents the quantitative evaluation of the registration accuracy of 20 patients measured in the standard diagnostic and the RT setup. Each registration was conducted using different (semi-) automatic registration tools offered by various commercial treatment planning systems, and compared to the clinical registration. To gain extensive insight into the evaluation, multiple evaluation methods were used, such as the segmentation-based, landmark-based, and dosimetric-based evaluation methods.

## 2 Materials and Methods

### 2.1 Patient acquisition

**Figure 1:**
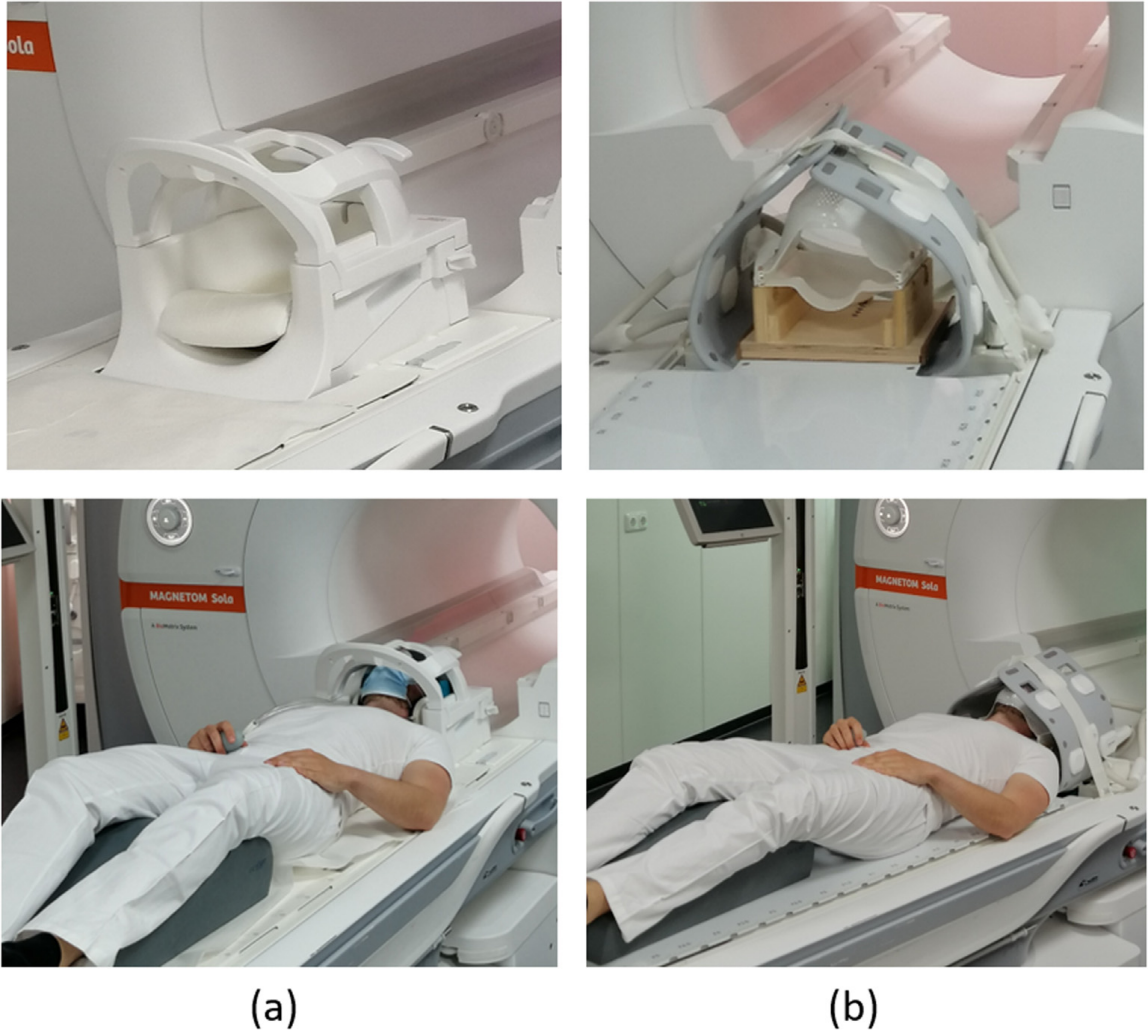
(a) The standard head coil (Head/Neck 20-channel coil) for MR-measurement in the diagnostic setup, and (b) the radiotherapy setup using an immobilization mask, a dedicated mask holder, an RT flat table top, and two 18-channel Ultraflex coils.

MRI scans of 20 head patients with brain tumors were acquired at the 1.5T MAGNETOM Sola (Siemens Healthineers, Erlangen, Germany) no longer than 5 days before treatment to minimize anatomical changes in patients [9]. Two different acquisition setups were employed: the diagnostic (MR_D_) and the radiotherapy setup (MR_RT_) consecutively (Fig. 1). In MR_D,_ the patients are measured in a standard head coil (Head/Neck 20-channel coil) as provided by the vendor, while in MR_RT_ the patients are positioned on an MR-compatible RT flat table top (INSIGHT system, Qfix, Avondale, USA) with a stereotactic mask immobilization system (Brainlab, Munich, Germany) and measured with two receiving coils (18-channels UltraFlex Large, Siemens Healthineers, Erlangen, Germany). As a part of standard clinical protocol, a contrast-enhanced T1-weighted Magnetization Prepared Rapid Gradient Echo (T1w MPRAGE, 1 mm isotropic) sequence is acquired (for detail see [16]). The planning CT (PCT, 1 × 1 × 1 mm^3^) is acquired at Somatom go.Open Pro (Siemens Healthineers, Erlangen, Germany).

### 2.2 Treatment planning

After imaging, MRI images were manually registered to the PCT as a part of clinical workflow using syngo.via RT Image Suite VB30 (Siemens Healthineers, Erlangen, Germany). Gross tumor volume (GTV)/CTV and PTV were defined on the MRI images, where CTV is identical to GTV in the clinical protocol for brain metastases. CTV-PTV margin ranges from 1 to 15 mm depending on the treatment option and dose concept. Treatment options include single-fraction SRS (18–21 Gy), fractionated stereotactic radiotherapy (FSRT, <12 fractions, total dose 18–48 Gy), and conventional radiation therapy (>12 fractions, total dose 45–56 Gy). The treatment option classification for this study is based on DEGRO and AAPM report on stereotactic radiosurgery [4], [20]. The OARs i.e., brainstem, chiasm, optical nerves, cochlea, acoustic nerve, and eyes were defined on the PCT, either using the automatic segmentation tool DirectORGANS (Siemens Healthineers, Erlangen, Germany) embedded in the CT-scanner or manually by physicians and verified by the responsible radiation oncologist.

### 2.3 Registration methods

Different MRI-CT registration methods were evaluated; the clinical registration (*R*_clin_) and the automatic registration (*R*_TPSx_) using three different tools: Raystation 8 (**TPS1**, Raysearch Laboratories, Stockholm, Sweden), syngo.via VB30 (**TPS2**, Siemens Healthineers, Erlangen, Germany), and Pinnacle 9.4 (**TPS3**, Phillips Radiation Oncology Systems, Fitchburg, WI) (see also Fig. 3). The clinical registration is typically done with MR_RT_, but MR_D_ can also be preferred in case the patient has received upfront resection; and when the registration between pre-and post-operative MRI is challenging. The clinical registration is done manually in **TPS2**, where both images are overlapped, manually adjusted iteratively, and visually inspected based on the brain contour. In our patient cohort, the MR_RT_ was used for the clinical registration of 15 patients, and the MR_D_ for 5 patients.

The automatic registrations were done for MR_D_ (*R*_TPSx,D_) and MR_RT_ (*R*_TPSx,RT_) to the PCT on each TPS. Optionally a region of interest (ROI) was defined (semi-automatic registrations), which included external geometry (for **TPS1**), the brain ventricles (for **TPS2**), or the entire brain volume (**TPS3**), as specifically recommended for each TPS by the respective manufacturer. For **TPS3**, the automatic registration was only feasible using ROI, and therefore no full-automatic registration was done for **TPS3**.

In total 11 registrations are available for each patient (1 clinical registration (R_clin. RT/D_ for either MR_D_ or MR_RT_), 2 full-automatic registrations (for both MR_D_ and MR_RT_), 3 semi-automatic registrations (for both MR_D_ and MR_RT_)). The semi-automatic registrations for TPS1 and TPS2 will be marked using a star symbol (TPS1* and TPS2*).

### 2.4 Segmentation-based registration accuracy evaluation

**Figure 2:**
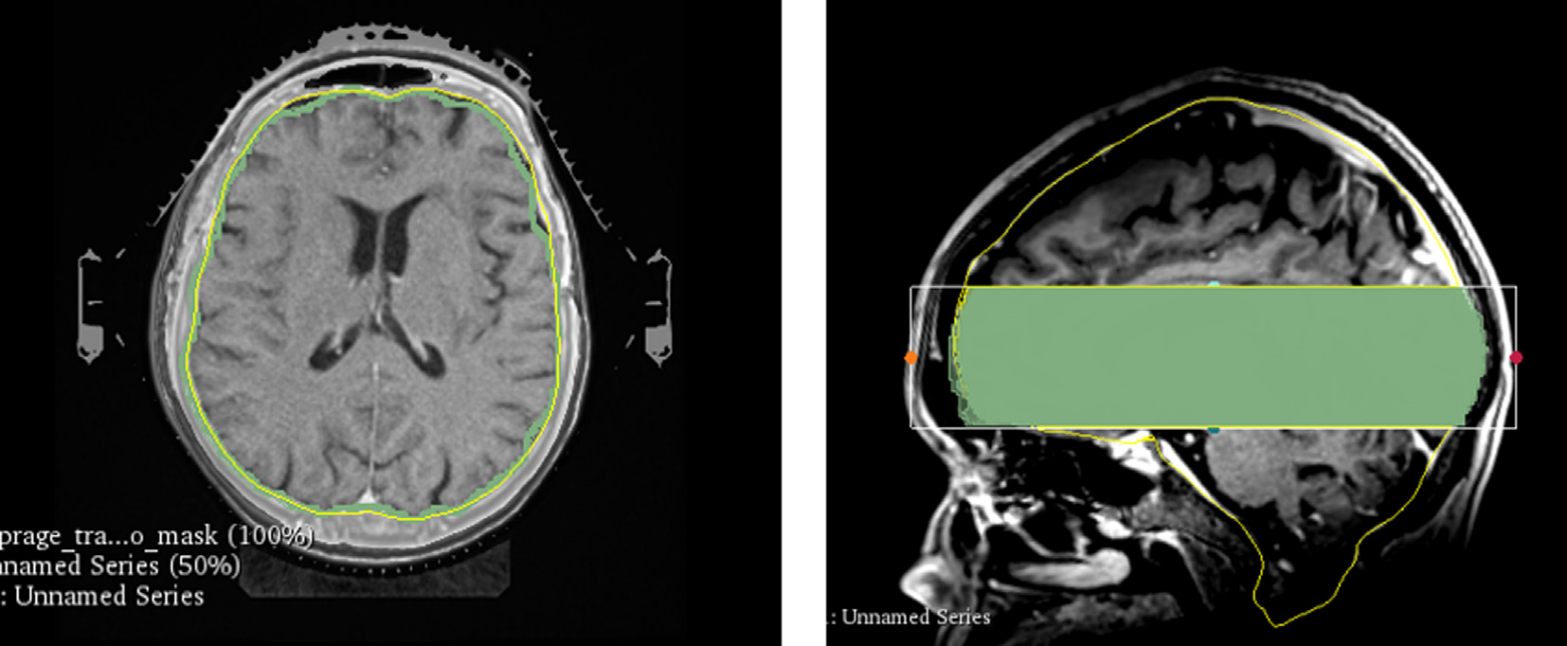
(a) A transversal view of the segmented brain of CT (yellow) and MRI (green), and (b) a sagittal view of the cropped brain segmentation used for segmentation-based evaluation. (For interpretation of the references to colour in this figure legend, the reader is referred to the web version of this article.)

Brain contour in MR_D_ and MR_RT_ was defined using the open-source brain extraction tool (HD-BET, [21]), whereas the brain contour of the CT was defined using an automatic model-based segmentation tool in **TPS1**. The segmentations were limited to a small area that excludes the most superior and inferior areas to avoid uncertainties coming from the brain contour differences between MRI and CT that are mostly present in these areas, and enable a consistent comparison excluding regions with reduced signal in the MRI (Fig. 2). The same axial range was cropped after the registration, where the most superior area starts in the middle of the brain, and the inferior area starts before the brainstem. The tumors are not necessarily located in the limited region. Dice similarity coefficient (DSC), mean Hausdorff distance (MHD), and the 95^th^ percentile of Hausdorff distance (HD_P95_) were used as evaluation metrics as recommended by the TG132 report [22]. All results for different registration metrics were evaluated using the open-source software 3Dslicer (version 4.10.2, [23]).

### 2.5 Landmark-based registration accuracy evaluation

Five common landmarks were defined on each MR_D_, MR_RT_, and PCT: (1) the tip of the alar cartilage; (2) the most inferior junction of the anterior wall of the sphenoid sinus with the sphenoid intersinus septum; (3) the confluence of the superior sagittal sinus and the transverse sinuses (MRI) and the internal occipital protruberence (CT); (4) the centroid of left eye; and (5) the centroid of right eye. These landmarks were chosen to ensure that rotational components in every direction were considered.

The mean Euclidean distance (*mEuD*) between the anatomical location of the landmark on the MRI after registration and the corresponding landmark location on CT was calculated.

### 2.6 Dosimetric evaluation of registration inaccuracies

**Figure 3:**
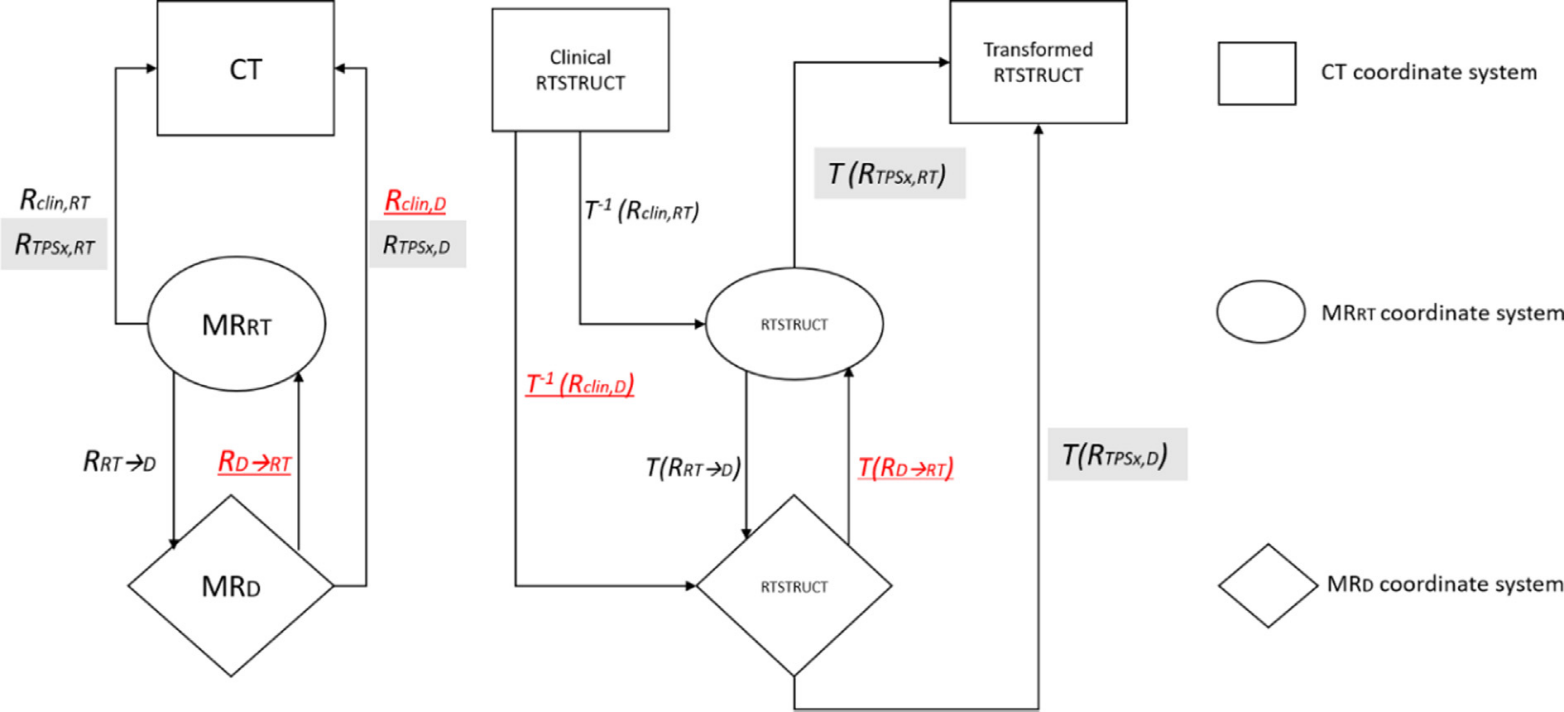
Flow diagram of the RTSTRUCT transformation between different coordinate systems (CT, MR_RT_, MR_D_) for the dosimetry-based evaluation method. Different font colors/attributes for the registration (R) and transformation (T) refer to two clinical cases: red+underlined = if **MR_D_** was used for clinical plan and; black = if **MR_RT_** was used for the clinical plan. Shown in grey boxes are fixed processes independent of the MR used for the clinical case. (For interpretation of the references to colour in this figure legend, the reader is referred to the web version of this article.)

A dose-volume-histogram (DVH) evaluation was conducted to assess the dosimetric impact of registration inaccuracies and was calculated for the GTV, PTV, and relevant OARs, i.e., brainstem, optical nerves, chiasma, eyes, and cochleas, using 3Dslicer. The OARs structures are only available on the clinical plan, hence only one set of MR has clinical structures. A recontouring was avoided to prevent intra- and interobserver variability in the delineation process, which can influence the evaluation accuracy. Therefore, the clinical OARs structures were inversely transformed back to its planning MRI (*R*_clin,RT_ or *R*_clin,D_) and then propagated onto the other MR set using registration metric *R*_RT_→_D_ or *R*_D_→_RT_ between MR_D_ and MR_RT_ using the Advanced Normalization Tool (ANTs) [24], [25] (see also Fig. 3). The structure set was then registered back onto the PCT using the obtained automatic registration metrics *R*_TPSx,RT_ or *R*_TPSx,D_, and the DVH of the shifted structures was recalculated using the clinical RT dose. V_80%_ was used to evaluate target coverage of SRS patients, who were prescribed to 80% isodose. Meanwhile, V_95%_ was used for the evaluation of most FSRT and conventional RT patients prescribed to 95% isodose. *D_max_* or *D_mean_* were obtained for each structure and compared to the clinical dose. Mean Percentage Error (*MPE*) was calculated for each OAR:

(2) $$\mathrm{MPE}=\frac{100\%}{n}\sum_{i=1}^{n}\frac{D_{\mathrm{reg},i}-D_{\mathrm{clin},i}}{D_{\mathrm{clin},i}}$$

where *D_reg_* is the *D_max_* or *D_mean_* received by OAR (Transformed RTSTRUCT, Fig. 3), *D_clin_* the clinical *D_max_*/*D_mean_*_._ (Clinical RTSTRUCT, Fig. 3), and *n* = *20* is the number of patients. Negative *MPE* implies that less dose is received by OAR compared to the clinical dose, and positive MPE implies more received dose. Wilcoxon signed-rank test was conducted on all evaluation metrics to determine the statistical significance of varying patient setup and registration methods (level of significance *p* < 0.05.)

**Figure 4:**
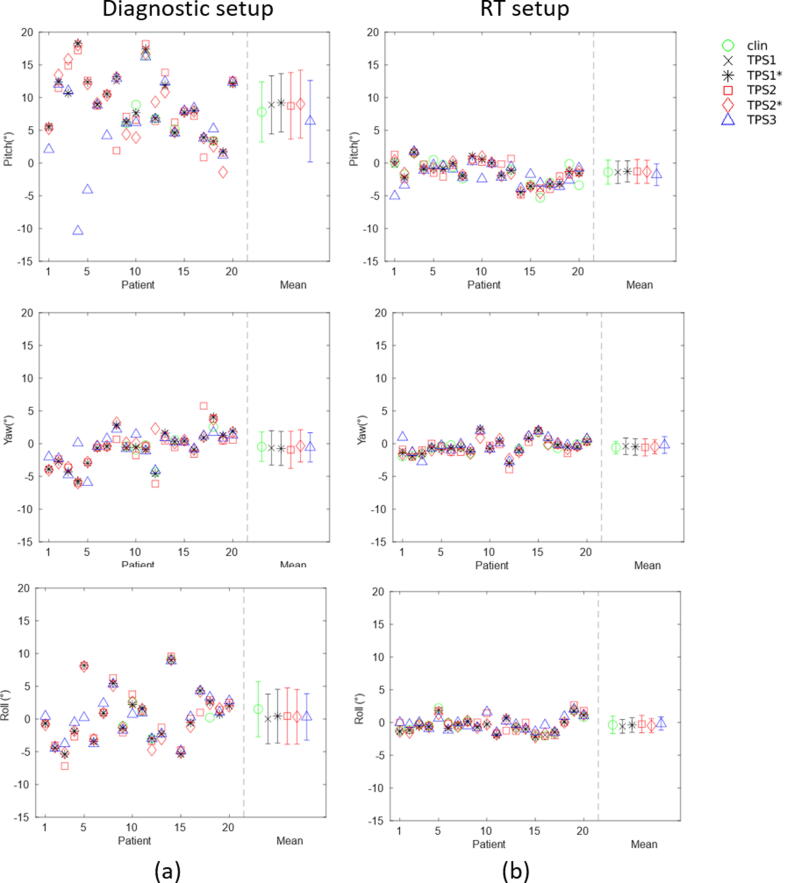
Rotation angle (roll-pitch-yaw) of MRI-CT rigid-registration, (a) MRI acquired in diagnostic setup, and (b) MRI acquired in radiotherapy setup.

For 20 patients, the rotation (pitch-yaw-roll) of all registration metrics was obtained (Fig. 4). The mean absolute pitch-yaw-roll angle across all TPSs in MR_D_ is 8.71° ± 5.07°/1.91° ± 2.49°/3.14° ± 3.92°, and in MR_RT_ 1.75° ± 1.36°/1.02° ± 0.75°/0.98° ± 0.68. Overall, the mean absolute angle and standard deviation were larger in MR_D_ than in MR_RT_.

## 3 Results

### 3.1 Segmentation-based registration accuracy evaluation

**Figure 5:**
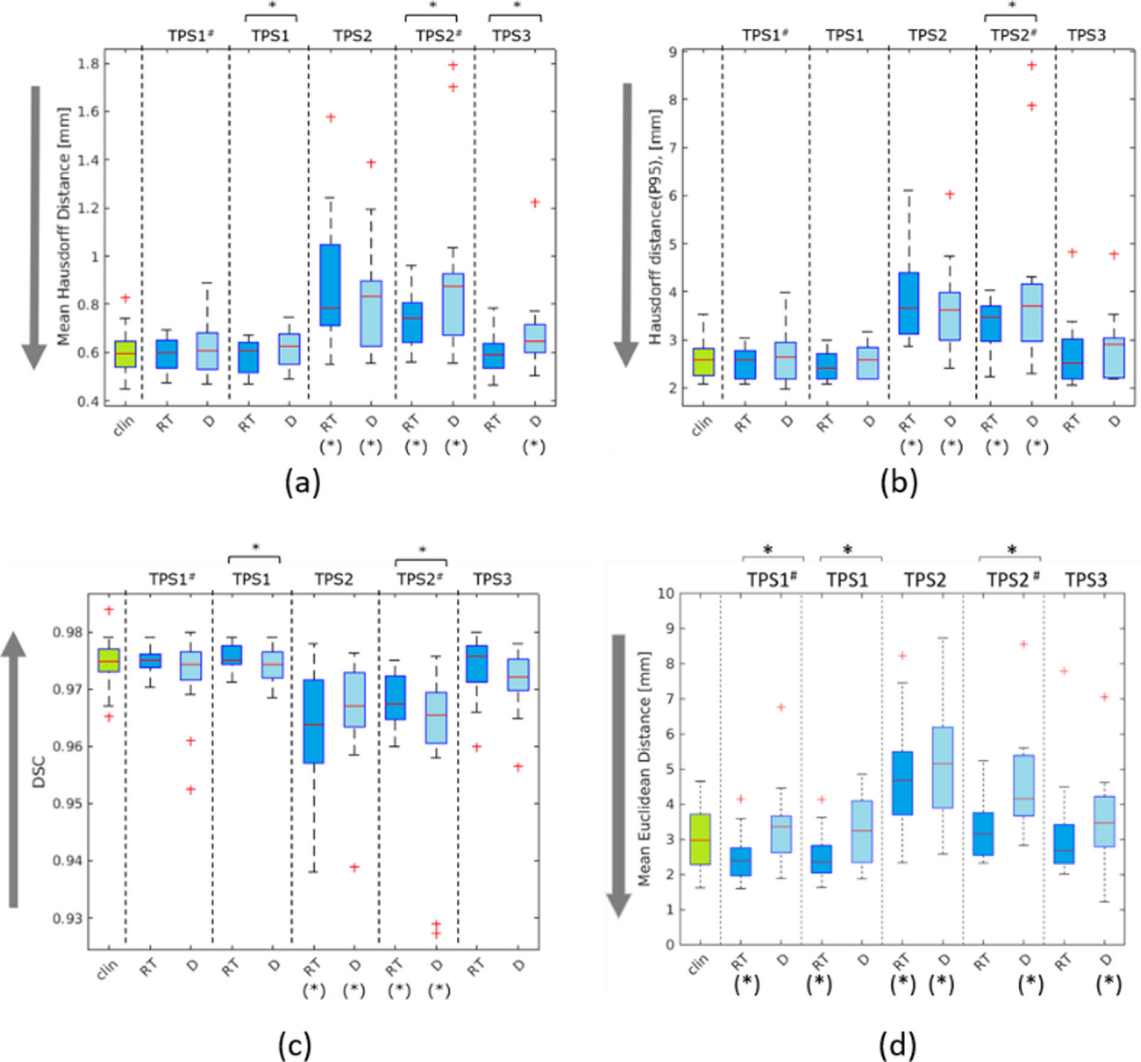
The results of the segmentation-based registration accuracy evaluation: (a) Mean Hausdorff distance, (b) 95th percentile of Hausdorff Distance, (c) Dice Similarity Coefficient, and the results of the landmarks-based registration accuracy evaluation: (d) Mean Euclidean Distance of landmarks across 3 different treatment planning platforms (^#^ with ROI) and 2 different MRI acquisition setups (RT: MRI acquisition in radiotherapy setup, D: MRI acquisition in diagnostic setup). Arrow direction points to better values. * on top of the graph indicates statistical significance between different setups within the same TPS, and (*) under the graph indicates statistical significance to the clinical registration (Wilcoxon signed-rank test, p<0.05).

The *MHD, HD_P95,_* and *DSC* of **TPS1** and TPS1* are not significantly different from the clinical registration for both diagnostic and RT setup (Fig. 5(a)–(c)). Nonetheless, clinical registrations have a wider distribution compared to **TPS1**. Similarly, the results of **TPS3** also do not differ from the clinical registration, except for the *MHD* of **TPS3_D_**. The registration accuracy of **TPS2** is the lowest regarding the magnitude and the width of the distribution and the results are significantly lower compared to the clinical registration for both setups.

Within the same TPS but different acquisition setups, TPS1*_D_ does not differ significantly compared to its TPS1*_RT_ counterpart (absolute difference of Δ*MHD* = 0.04 mm, Δ*HD_P95_* = 0.07 mm). Meanwhile, with TPS2* registration, a significant improvement to the registration accuracy was observed in TPS2*_RT_ compared to TPS2*_D_ (absolute difference of Δ*MHD* = 0.16 mm, Δ*HD_P95_* = 0.64 mm). For **TPS3**, a significant difference can be seen based on *MHD*, however not based on *HD_P95_* and *DSC.* The difference in mean *MHD* between the best (**TPS1_RT_)** and the worst registration (TPS2*_D_) is 0.3 mm. The use of an ROI in the automatic registration did not significantly improve the registration accuracy, except for **TPS2_RT_**.

### 3.2 Mean Euclidean Distance of Landmarks

Fig. 5(d) shows the *mEuD* of the five defined landmarks. The best registration was delivered by TPS1*_RT_ (2.49 ± 0.68 mm) and **TPS1_RT_** (2.56 ± 0.70 mm), and the worst registration was delivered by **TPS2_D_** (5.18 ± 1.73 mm) and TPS2*_D_ (5.86 ± 5.00 mm). Significant improvement by using RT setup was observed in TPS1*, **TPS1**, and TPS2*. Within TPS2* registration, RT setup improves the mean *mEUD* by 2.65 mm. The use of an ROI did not show a significant improvement, except for **TPS2_RT_**.

**Figure 6:**
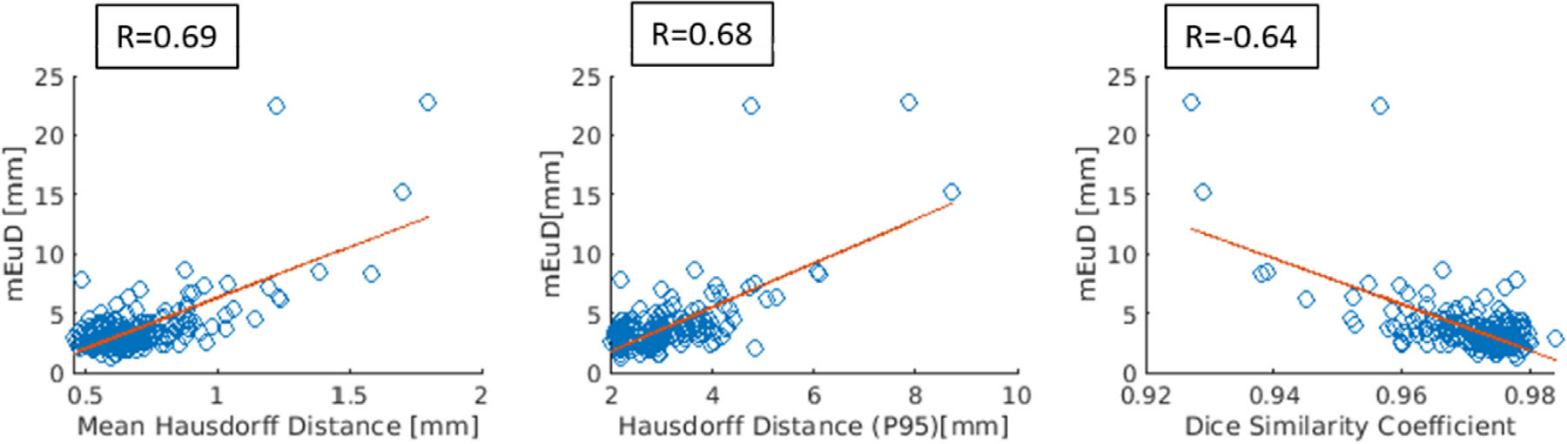
Correlation matrix between mean Euclidean Distance (*mEuD*), mean Hausdorff Distance, 95^th^ percentile of the Hausdorff Distance, and Dice Similarity Coefficient. Data points are linear fitted, where R is the correlation coefficient.

Fig. 6 shows the correlation matrix between the *mEuD* and the *MHD, HD_P95_*, and DSC, where a strong correlation between the *mEUD* and these metrics was observed.

### 3.3 Dosimetric evaluation of registration inaccuracies

**Figure 7:**
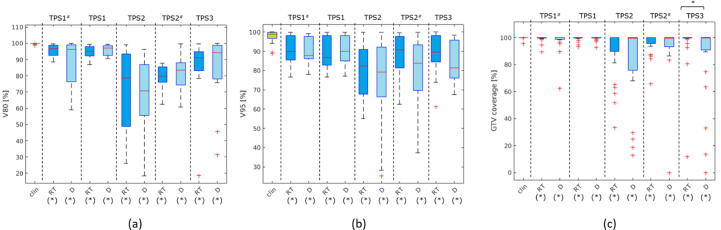
Box plots of dosimetric evaluation of MRI-CT registrations: (a) V_80%_ of patients prescribed to 80% isodose (b) V_95%_ of patients prescribed to 95% isodose, and (c) GTV coverage: across 3 different treatment planning platforms (^#^ with ROI) and 2 different patient acquisition (RT: MRI acquisition in radiotherapy setup, D: MRI acquisition in diagnostic setup.). * on top of the graph indicates statistical significance between different setups within the same TPS, and (*) under the graph indicates statistical significance to the clinical registration (Wilcoxon signed-rank test, p < 0.05).

In six SRT patients, RT was prescribed to the 80% isodose, from which five were treated with SRS and one with FSRT. Mean V80% for automatic registration in MR_RT_ varied between 73 ± 24.87% (**TPS2**) and 95.2 ± 3.82% (TPS1*) (Fig.  7(a)), while in MR_D_, mean V_80%_ varied between 67 ± 25.19% (**TPS2**) and 96 ± 3.25% (**TPS1**).

Fourteen patients were prescribed to 95% isodose. Mean V_95%_ in MR_RT_ varied between 79.8 ± 14.51% (**TPS2**) and 91 ± 7.29% (TPS1*) (Fig.  7(b)), while in MR_D_ it varied between 74.4 ± 23.35% (**TPS2**) and 90.9 ± 7.35% (**TPS1**).

In MR_RT_, mean GTV coverage varied between 91.5 ± 16.6%. (**TPS2**) and 99.2 ± 1.87% (TPS1*), while in MR_D_, it varied between 83.2 ± 27.46% (**TPS2**), and 99.4 ± 1.49% (TPS1*) (Fig.  7(c)).

The various registration methods influenced the clinical DVH significantly. RT setup improves PTV coverage when using **TPS2**, but does not improve PTV coverage when using **TPS1**. GTV coverage is improved when using RT setup, and especially significant for **TPS3**, where results of **TPS3_RT_** are significantly better than **TPS3_D_**. Overall, the smallest variation in the V80%, V95%, and GTV coverage for automatic registration was delivered by **TPS1,** and the largest variation was delivered by **TPS2**.

**Figure 8:**
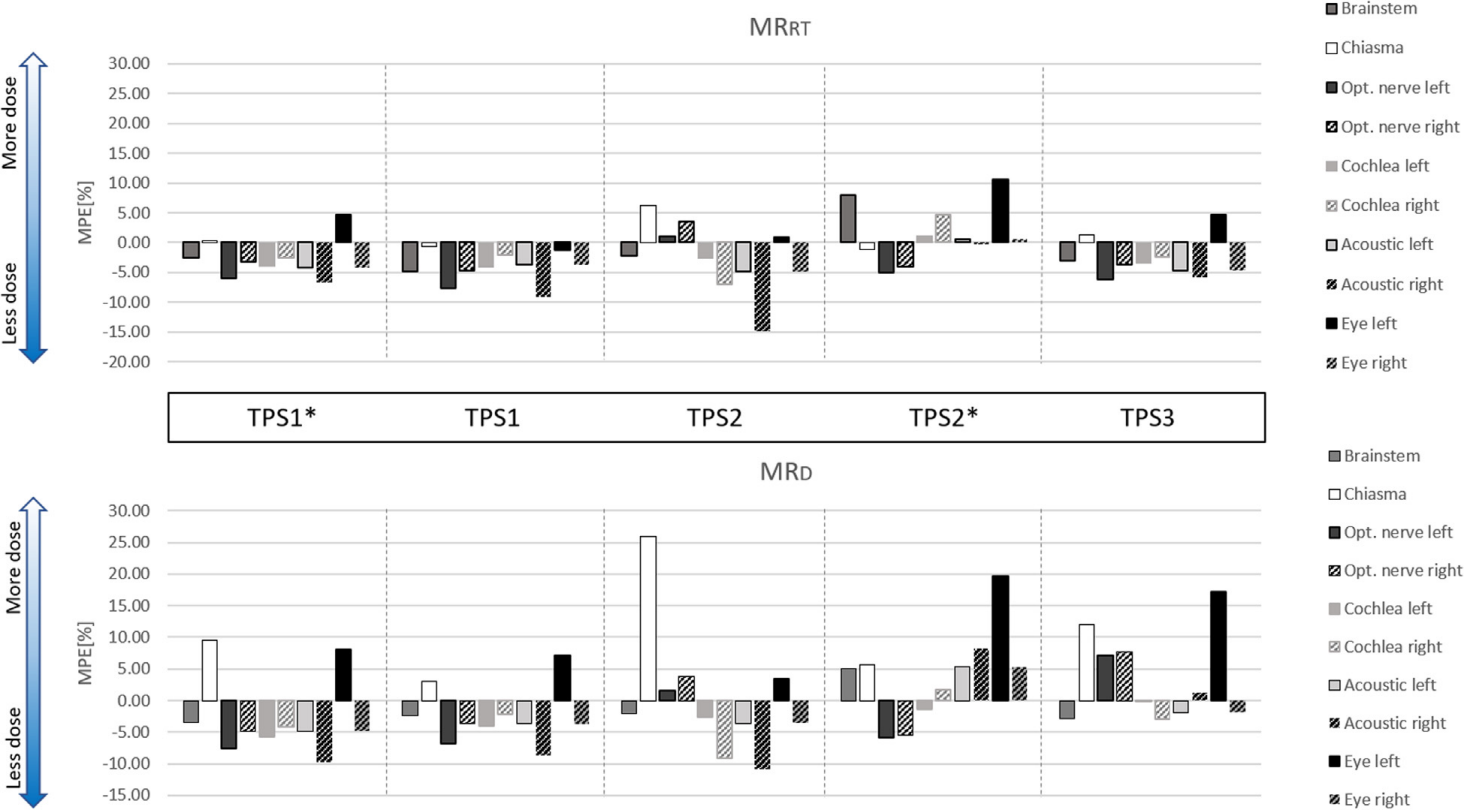
Mean percentage error (MPE) of D_max_ and D_mean_ of OARs registered using various treatment planning systems (TPS) compared to clinical registration. (MR_RT_: MR acquisition in radiotherapy setup, MR_D_: MR acquisition in diagnostic setup.)

Mean percentage error (*MPE*) was calculated for each OAR (Fig. 8). The choice of acquisition setup and registration method influence the *D_max_/D_mean_* of each OAR. The largest variation was found in chiasma (*MPE* = −1% to 26%, *D_ma_*_x_ = 52 Gy) and the right acoustic nerve (*MPE* = −1.3% to 20%, *D_max_* = 51 Gy, *D_mean_* = 20 Gy); while the smallest variation was found in left cochlea (*MPE* = −6% to 1.1 %, *D_max_* = 51 Gy, *D_mean_* = 20 Gy). Despite this considerable variation, the clinical dose limits were, however, in none of the cases exceeded.

## 4 Discussion

Mean absolute rotation angle for MRI-CT registration in pitch, yaw, and roll direction were evaluated for MR_RT_ and MR_D_. Brunt et al. reported that patient positioning in either the MR_D_ or MR_RT_ results in a different head extension [11]. Nonetheless, to this date, there has been no extensive multi-metric study on the influence of MRI acquisition setup which is comparable to the methodology presented in this work. Based on our quantitative results, the larger mean absolute rotation angle in MR_D_-CT registration compared to MR_RT_-CT rotation indicates that the head extension in RT setup is more similar to the patient’s head position during CT acquisition and irradiation. The overall larger standard deviation of the rotation in MR_D_ also indicates that a higher reproducibility of head positioning in the MR_RT_ setup can be achieved. Nonetheless, minimal rotational uncertainties in patient fixation using a stereotactic head mask system might still be present [26].

The results from the segmentation-based and landmark-based evaluation methods lead to the conclusion that **TPS1** (Raystation v8.0) is the best automatic registration method while **TPS2** (syngo.via VB30) is the least optimal automatic registration method (see Section 3.1). The registration results of **TPS1** (in both MR setups) and **TPS3_RT_** are comparable or better than the clinical registrations, while a significant difference to the clinical registration was found using **TPS2** (both MR setups) and **TPS3_D_**. When comparing the registration quality in both MR_RT_ and MR_D_ using **TPS1**, the acquisition setup did not show a significant impact. However, when choosing a less optimal registration method such as TPS2*, the use of MR_RT_ setup significantly improved the registration. The results of **TPS2** are significantly lower compared to the clinical registration, even though both were done in the same TPS. Nonetheless, a more meticulous manual adjustment was done in the clinical registration. This implies that the automatic adjustment is significantly improved by manual adjustments by experts.

The use of segmentation- and voxel-intensity-based evaluation for registration accuracy has been addressed as having severe limitations by Rohlfing et al. for non-rigid image registration [27]. For rigid registration, using the Target Registration Error (TRE) of a set of landmarks is sufficient and is widely accepted as the gold standard [28]. It is, however, difficult to find common landmarks on CT and MRI in particular in the case of retrospective studies based on the available clinical data. For the segmentation-based evaluation of the MRI-CT rigid registration in this study, we chose with the brain a structure that is feasible to define on both images. Disadvantageous is, however, that this structure is rather large and thus does not provide the advantage of smaller, more localized ROIs when used for image overlap evaluation based on the DSC [27]. By using both landmarks- and segmentation-based metrics for the evaluation, the registration accuracy across the different MRI acquisition setups and registration methods can be investigated in various ways and the quality of the evaluation can be ensured. In this study, a strong correlation was found between the calculated *mEUD* and the segmentation-based evaluation metrics. Due to the lack of common landmarks or ROIs in the infratentorial region, the evaluation of this exclusive area is not part of this study but would be a critical point to address in future studies.

Automatic registration using **TPS1** delivered a comparable or better registration accuracy compared to the clinical registration regarding segmentation and landmark-based evaluation. However, even when a better registration accuracy was achieved, PTV and GTV coverages were lower than the clinical ones. Considering that the dose distribution and tumor coverages used for evaluation were only optimized on the tumor position that was derived from MRI images using the clinical registration; this variation of tumor coverage is to be expected and does not imply that registration using **TPS1** is inferior to the clinical registration. This also explains the low variances of the clinical registration. Overall, different registration methods lead to variations in the DVH. *MPE* of 26% was found in chiasma, which indicates that registration inaccuracy could have fatal consequences for OARs, especially in cases where OARs receive a high dose close to the clinical dose limit, as then the dose limit will be exceeded. This can occur, for example, when the GTV lies in close proximity to or in contact with the OAR. The RT setup improves the PTV coverage when using **TPS2**, which offers the least optimal registration method, and brings a significant improvement to GTV coverage, especially in **TPS3.** Hence it can be concluded, that the results based on GTV coverage agree with the segmentation-based evaluation results indicating that RT setup improves the registration accuracy.

Compared to other studies observing different regions such as the head and neck [14], and pelvis [13], the influence of the acquisition setup in brain tumor patients is reduced. A study conducted by Nagtegaal et al. [29] stated that an immobilization mask does not bring additional value to the registration in stereotactic radiotherapy of brain tumors. However, in their study, the MRI-CT rigid registration was done based on a single normalized mutual information-based registration algorithm by Bol et al. [30] and it did not cover the registration across different registration methods, which in this study has been proven to have a large influence on the registration accuracy. Meanwhile, other studies evaluating the accuracy of MRI-CT registration of the head did not compare different MRI acquisition setups [18], [19].

The acquisition in an MR_RT_ setup offers other advantages such as fewer motion artifacts and anatomical differences to CT images in comparison to MR_D_ [9]. Additionally, the use of an immobilization mask improves the reproducibility of the patient’s localization, which consequentially improves treatment delivery [31], [32]. We showed using a wide variety of metrics that RT setup improves the registration accuracy when using a certain automatic registration method, although the influence of the registration method is more significant than the acquisition setup.

Based on our results, optimal automatic registration seems to be the best registration approach providing reduced interobserver variability and similar or even superior accuracy to manual registration, with the added benefit of reduced manual registration time. We showed that using the automatic registration in Raystation in combination with an MRI acquired in RT setup provided the most optimal registration, resulting in smaller variation in the head rotation, where all evaluation metrics showed similar or better values than the clinical registration that relies on user-intervention within syngo.via. Additionally, OARs received less dose while still maintaining good GTV coverage. In any case, such automatic registration methods need to be quality assured by an experienced radiation oncologist if used in clinical practice.

## 5 Conclusions

In this study, we showed that a dedicated RT setup during MRI acquisition ensures a similar head position as during treatment. Acquiring patients in RT setup adds value to the MRI-CT registration for the brain RT when using an automatic registration tool that is insufficient. When an optimal registration method is used, RT setup does not improve the registration significantly, but still delivers smaller variation in the head rotation compared to the diagnostic setup. Both the registration method and the acquisition setup affect the DVHs of RT planning.

## Declaration of Competing Interest

Universitätsklinikum Erlangen, Strahlenklinik and Siemens Healthineers (Erlangen, Germany) have institutional research contracts not related to this specific work. Christoph Bert and Florian Putz act as speakers in training courses of Siemens Healthineers.
